# Service readiness of health facilities in Bangladesh, Haiti, Kenya, Malawi, Namibia, Nepal, Rwanda, Senegal, Uganda and the United Republic of Tanzania 

**DOI:** 10.2471/BLT.17.191916

**Published:** 2017-09-05

**Authors:** Hannah H Leslie, Donna Spiegelman, Xin Zhou, Margaret E Kruk

**Affiliations:** a Department of Global Health and Population, Harvard TH Chan School of Public Health, 677 Huntington Avenue, Boston, MA 02115, United States of America (USA).; b Department of Epidemiology, Harvard TH Chan School of Public Health, Boston, USA.

## Abstract

**Objective:**

To evaluate the service readiness of health facilities in Bangladesh, Haiti, Kenya, Malawi, Namibia, Nepal, Rwanda, Senegal, Uganda and the United Republic of Tanzania.

**Methods:**

Using existing data from service provision assessments of the health systems of the 10 study countries, we calculated a service readiness index for each of 8443 health facilities. This index represents the percentage availability of 50 items that the World Health Organization considers essential for providing health care. For our analysis we used 37–49 of the items on the list. We used linear regression to assess the independent explanatory power of four national and four facility-level characteristics on reported service readiness.

**Findings:**

The mean values for the service readiness index were 77% for the 636 hospitals and 52% for the 7807 health centres/clinics. Deficiencies in medications and diagnostic capacity were particularly common. The readiness index varied more between hospitals and health centres/clinics in the same country than between them. There was weak correlation between national factors related to health financing and the readiness index.

**Conclusion:**

Most health facilities in our study countries were insufficiently equipped to provide basic clinical care. If countries are to bolster health-system capacity towards achieving universal coverage, more attention needs to be given to within-country inequities.

## Résumé

**Objectif:**

Évaluer la disponibilité des services dans les établissements de santé du Bangladesh, d'Haïti, du Kenya, du Malawi, de Namibie, du Népal, d'Ouganda, de République-Unie de Tanzanie, du Rwanda et du Sénégal.

**Méthodes:**

En nous appuyant sur les données existantes tirées de l'évaluation de la prestation des services dans les systèmes de santé des 10 pays étudiés, nous avons calculé un indice de disponibilité des services pour chacun des 8443 établissements de santé. Cet indice correspond à la disponibilité en pourcentage de 50 éléments que l'Organisation mondiale de la Santé estime essentiels pour assurer les soins de santé. Dans le cadre de notre analyse, nous avons utilisé entre 37 et 49 éléments de la liste. Nous avons eu recours à une régression linéaire pour évaluer le pouvoir explicatif indépendant de quatre caractéristiques nationales et quatre caractéristiques au niveau des établissements concernant la disponibilité des services établie.

**Résultats:**

Les valeurs moyennes de l'indice de disponibilité des services étaient de 77% pour les 636 hôpitaux et de 52% pour les 7807 centres de santé/dispensaires. L'analyse a révélé des insuffisances particulièrement courantes en matière de médicaments et de capacités de diagnostic. L'indice de disponibilité variait davantage entre les hôpitaux et les centres de santé/dispensaires d'un même pays qu'entre différents pays. Une faible corrélation a été constatée entre les facteurs nationaux liés au financement de la santé et l'indice de disponibilité.

**Conclusion:**

La plupart des établissements de santé des pays étudiés n'étaient pas dotés d'équipements suffisants pour prodiguer les soins cliniques de base. Il est nécessaire de prêter davantage attention aux inégalités au sein des pays pour qu'ils renforcent les capacités de leur système de santé en vue d'assurer une couverture universelle.

## Resumen

**Objetivo:**

Evaluar la disponibilidad del servicio de los centros sanitarios en Bangladesh, Haití, Kenya, Malawi, Namibia, Nepal, la República Unida de Tanzanía, Rwanda, Senegal y Uganda.

**Métodos:**

Usando los datos existentes de las evaluaciones sobre prestación de servicios de sistemas sanitarios de los 10 países de estudio, se ha calculado un índice de disponibilidad del servicio para cada uno de los 8443 centros sanitarios. El índice representa el porcentaje de disponibilidad de 50 elementos que la Organización Mundial de la Salud considera esenciales para proporcionar atención sanitaria. Para el análisis, se han utilizado entre 37 y 49 de los elementos de la lista. Se ha utilizado la regresión lineal para evaluar el poder independiente descriptivo de cuatro características nacionales y cuatro a nivel del centro sobre la disponibilidad del servicio registrado.

**Resultados:**

Los valores medios del índice de la disponibilidad del servicio fueron del 77% para los 636 hospitales y del 52% para los 7807 centros de salud/clínicas. Las deficiencias en los medicamentos y la capacidad de diagnóstico fueron particularmente comunes. El índice de disponibilidad varió más entre hospitales y centros de salud/clínicas en el mismo país que entre países. Existe una correlación débil entre los factores nacionales relacionados con la financiación sanitaria y el índice de disponibilidad.

**Conclusión:**

La mayoría de los centros sanitarios en nuestros países de estudio fueron equipados de forma insuficiente para proporcionar atención sanitaria básica. Si los países van a reforzar la capacidad del sistema sanitario hasta conseguir la cobertura universal, se necesita poner más atención a las desigualdades dentro del país.

## ملخص

**الغرض:**

تقييم جاهزية خدمات المرافق الصحية في بنغلاديش وهايتي وكينيا وملاوي وناميبيا ونيبال ورواندا والسنغال وأوغندا وجمهورية تنزانيا المتحدة.

**الطريقة:**

باستخدام البيانات المتاحة من واقع تقييمات تقديم خدمات أنظمة الصحة في بلدان الدراسة العشرة (10)، قمنا بحساب مؤشر جاهزية الخدمة لكل من المرافق الصحية البالغ عددها 8443 مرفقًا. ويمثل هذا المؤشر نسبة التوفر المئوية لخمسين عنصرًا تعتبرها منظمة الصحة العالمية ضرورية لتوفير الرعاية الصحية. واستخدمنا 37-49 من العناصر المدرجة في القائمة لإجراء التحليل الصادر عنا. وقد استخدمنا أسلوب التحوّف الخطي لتقييم القدرة التوضيحية المستقلة لأربع خصائص وطنية وعلى مستوى المرافق الصحية فيما يتعلق بجاهزية الخدمات الواردة في التقارير.

**النتائج:**

بلغت القيم المتوسطة لمؤشر جاهزية الخدمة 77‏٪ لعدد 636 مستشفى و‏52‏٪ لعدد 7807 مراكز صحية وعيادات. وكانت أوجه القصور شائعة في الأدوية والقدرة التشخيصية بوجه خاص. وظهر تباين في مؤشر الجاهزية بشكل أكبر بين المستشفيات والمراكز الصحية/العيادات الموجودة في نفس البلد أكثر مما بينها وبين مثيلاتها في البلدان الأخرى. وكان هناك ارتباط ضعيف بين العوامل الوطنية المتصلة بتمويل الصحة ومؤشر الجاهزية.

**الاستنتاج:**

لم تكن معظم المرافق الصحية في بلدان دراستنا مجهزة تجهيزًا كافيًا لتوفير الرعاية السريرية الأساسية. وإذا ما أرادت البلدان تعزيز قدرة النظام الصحي على تحقيق التغطية الشاملة، فإنه ينبغي إيلاء مزيد من الاهتمام لحالات التفاوت داخل البلدان **. **

## 摘要

**目的:**

旨在评估海地、肯尼亚、卢旺达、马拉维、孟加拉国、纳米比亚、尼泊尔、塞内加尔、坦桑尼亚共和国和乌干达卫生机构服务配备状态。

**方法:**

运用从这 10 个研究对象国卫生系统服务提供评估机构提供的现有数据，我们对 8443 个卫生机构的服务配备指数进行了计算。该指数代表 50 个由世界卫生组织认定的医疗保健必要项目的可用情况。 我们选取其中 37–49 个项目进行分析。我们采用线性回归法对服务配备报告上的四个国家级和四个机构级特征的独立阐释能力进行了评估。

**结果:**

其中 636 家医院的服务配备指数平均数是 77%，且 7807 家医疗中心/诊所的平均数为 52%。给药和诊断能力不足尤为普遍。相同国家医院和医疗中心/诊所间的配备指数差异比不同国家间的相应配备指数差异更大。与卫生筹资相关的国家因素和配备指数之间相关性较弱。

**结论:**

我们的研究对象国中，大多数国家的卫生机构在提供基本卫生护理方面配备不足。如果这些国家计划提升卫生系统能力，以实现全民卫生服务，它们需要多关注国内的不公平现象。

## Резюме

**Цель:**

Оценить степень готовности медицинских учреждений к оказанию услуг в Бангладеш, Гаити, Кении, Малави, Намибии, Непале, Объединенной Республике Танзания, Руанде, Сенегале и Уганде.

**Методы:**

Используя имеющиеся данные, полученные из оценок предоставления медицинского обслуживания системами здравоохранения в 10 исследуемых странах, авторы рассчитали индекс готовности к оказанию услуг для каждого из 8443 медицинских учреждений. Этот индекс представляет собой процентный показатель наличия 50 пунктов, которые Всемирная организация здравоохранения считает необходимыми для оказания медицинской помощи. Для анализа авторы использовали от 37 до 49 пунктов из этого списка. Авторы использовали линейную регрессию для оценки степени влияния независимых показателей — четырех характеристик на национальном уровне и четырех характеристик на уровне медицинского учреждения — на заявленную степень готовности к оказанию услуг.

**Результаты:**

Средние значения индекса готовности к оказанию услуг составили 77% для 636 больниц и 52% для 7807 медицинских центров и клиник. Особенно был распространен недостаток медикаментов и диагностического потенциала. Индекс готовности больше варьировался между больницами и медицинскими центрами/клиниками внутри одной страны, чем между разными странами. Наблюдалась слабая корреляция между национальными факторами, связанными с финансированием здравоохранения, и индексом готовности.

**Вывод:**

Большинство медицинских учреждений в исследуемых странах были недостаточно подготовлены для оказания базовой клинической помощи. Если страны будут наращивать потенциал системы здравоохранения в целях обеспечения всеобщего охвата, им следует больше внимания уделять неравенству внутри страны.

## Introduction

In adopting the sustainable development goals (SDGs) in September 2015, global governments and multilateral organizations endorsed universal health coverage as both a critical element of sustainable development and a prerequisite for achieving equity in global health.^1^ If the goal of universal coverage is to be achieved, we will need health-system financing that is adequate and sustainable and health services that are accessible and effective. While research from multiple fields has provided insights on health-system design^2^ and the utilization of care,^3^ there has been relatively little investigation of the capacity of health facilities to provide essential services.

Service readiness – a subset of the structural quality of care in the Donabedian triad of structure, process and outcome^4^ – is a prerequisite to the delivery of quality health care.^5^ During the era of the Millennium Development Goals, the capacity of health facilities to provide care and the quality of the care delivered received far less scrutiny than access to care alone.^5^ The resultant deficit must be addressed if the SDGs are to be achieved.^6^ Although there have been many area-specific studies on the capacity of health facilities to provide disease-specific services,^7^^–^^11^ there have only been a few comparative and multicountry studies of general service readiness.^12^^,^^13^ Between 2003 and 2013, recognizing the need for a comparable metric of health facility capacity, the World Health Organization (WHO) developed a general service readiness index.^14^ In 2013, this index was updated to cover 50 items: seven basic amenities such as water and power, six types of basic equipment such as stethoscopes, nine infection prevention measures such as the availability of gloves, eight types of diagnostic test and 20 essential medications – including drugs for infectious as well as noncommunicable diseases (Table 1).^15^ In representing the percentage of these items that are readily available in health facilities, the service readiness index is intended to capture overall capacity to provide the general health services that should be available in all facilities – whether they be at primary, secondary or tertiary level. It does not include indicators for hospital-specific readiness.^15^

**Table 1:** Sample size for each item used in the evaluation of service readiness indexes, 10 countries, 2007–2015

Domains and items	No. of facilities			
	With valid data	With non-systematically missing data**^a^**	With systematically missing data	
			Item not investigated in some facilities**^b^**	Item not investigated in whole national assessment
**Basic amenities**				
Electricity – i.e. uninterrupted power source or functional generator with fuel	8443	0	0	0
Safe water – i.e. improved source within 500 m of the facility	8424	19	0	0
Exam room with auditory and visual privacy	8433	10	0	0
Client sanitation facilities	7972	60	0	411^c^
Communication equipment – i.e. functional phone or shortwave radio	8436	7	0	0
Computer with email and internet	8443	0	0	0
Emergency transportation	8441	2	0	0
**Basic equipment**				
Adult scale	7981	12	450	0
Paediatric scale	8261	6	176	0
Thermometer	8263	4	176	0
Stethoscope	8214	1	228	0
Blood pressure apparatus	8214	1	228	0
Light source	8226	2	215	0
**Infection prevention**				
Safe final disposal of sharps	8218	161	64	0
Safe final disposal of infectious waste	8287	107	49	0
Sharps box/container in exam room	8443	0	0	0
Waste bin with lid and liner in exam room	8443	0	0	0
Surface disinfectant	8443	0	0	0
Single-use standard disposable or auto-disposable syringes	8443	0	0	0
Soap and running water or alcohol-based hand sanitizer	8443	0	0	0
Latex gloves	8443	0	0	0
Guidelines for standard precautions against infection	8443	0	0	0
**Diagnostic capacity**				
Haemoglobin test	5470	3	2970	0
Blood glucose test	5470	3	2970	0
Malaria diagnostic capacity	5043	3	1849	1548^d^
Urine dipstick for protein	5470	3	2970	0
Urine dipstick for glucose	5470	3	2970	0
HIV diagnostic capacity	5043	3	1849	1548^d^
Syphilis rapid diagnostic test	5472	1	2970	0
Urine pregnancy test	5470	3	2970	0
**Medication**				
Amitriptyline tablet	6114	0	194	2135^e^
Amlodipine tablet or alternative calcium channel blocker	6114	0	194	2135^e^
Amoxicillin syrup/suspension or dispersible tablet	8106	2	335	0
Amoxicillin tablet	8106	2	335	0
Ampicillin powder for injection	8108	0	335	0
Beclometasone inhaler	6114	0	194	2135^e^
Ceftriaxone injection	8104	4	335	0
Enalapril tablet or alternative ACE inhibitor	6114	0	194	2135^e^
Fluoxetine tablet	0	0	0	7480^f^
Gentamicin injection	8104	4	335	0
Glibenclamide tablet	6114	0	194	2135^e^
Ibuprofen tablet	5378	4	241	2820^g^
Insulin injection	6114	0	194	2135^e^
Metformin tablet	6114	0	194	2135^e^
Omeprazole tablet or alternative – e.g. pantoprazole or rabeprazole	6114	0	194	2135^e^
Oral rehydration solution	8106	2	335	0
Paracetamol tablet	8105	3	335	0
Salbutamol inhaler	6114	0	194	2135^e^
Simvastatin tablet or other statin – e.g. atorvastatin, pravastatin or fluvastatin	6114	0	194	2135^e^
Zinc sulfate tablet or syrup	6114	0	194	2135^e^

ACE: angiotensin converting enzyme; HIV: human immunodeficiency virus.

^a^ Item was included but no valid response recorded for a given facility.

^b^ Item was included in national service provision assessment but only investigated if the facility offered the relevant clinical service.

^c^ Missing from data for Namibia.

^d^ Missing from data for Bangladesh.

^e^ Missing from data for Kenya, Namibia, Rwanda and Uganda.

^f^ Not asked in any country. Since fluoxetine had not been assessed in any of the service provision assessments used as our data sources, we excluded it from our analyses.

^g^ Missing from data for Haiti, Senegal and United Republic of Tanzania.

The general service readiness index is increasingly being used in subnational or national assessments^16^^,^^17^ and has also been adapted for disease-specific studies.^18^ There appears to have been only one published analysis of the service readiness index across multiple countries: a comparison of the readiness of health facilities in six countries, which identified major gaps.^14^ Since 2013, when the latter comparison was published, the content of the service readiness index has been updated to incorporate basic capacity to address noncommunicable diseases^15^ and nationally representative assessments of health systems have been conducted in multiple countries.

In analyses of facility readiness, the use of a generalizable metric such as the service readiness index is important for several reasons. For example, such a metric can indicate the capacity of facilities to provide essential care, including their ability to respond to traditional and emerging health challenges.^19^ Use of such a metric enables the identification of within-country differences that may suggest inequities in resource distribution and can provide a basis for comparing the efficiency of health systems in translating financial inputs into readiness to meet population health needs. Theorists interested in health-system reform have classified the causal determinants of health-system performance into five main areas: behaviour, financing, organization, payment and regulation.^20^ Changes in any of these areas should affect the accessibility, efficiency and quality of health care and, ultimately, population health.^20^ In low- and middle-income countries, positive – though variable – associations between total health expenditure and population health have been observed.^21^ In addition, external donor assistance for health has been linked with reduced mortality^22^^,^^23^ whereas greater reliance on individual out-of-pocket expenditure has been associated with lower health coverage.^2^ Despite these observations, relatively little is known about the relationship between investigated determinants of health-system performance and the capacity of health systems to deliver care.

The objective of the study was to evaluate the service readiness of health facilities in Bangladesh, Haiti, Kenya, Malawi, Namibia, Nepal, Rwanda, Senegal, Uganda and the United Republic of Tanzania. 

## Methods

### 

#### Study sample

The study sample comprised the health facilities that had been included in service provision assessments by the Demographic and Health Survey (DHS) programme between 2007 and 2015.^24^ Assessment data were available in January 2017 for 10 low- and middle-income countries: Bangladesh, Haiti, Kenya, Malawi, Namibia, Nepal, Rwanda, Senegal, Uganda and the United Republic of Tanzania. 

The data from Kenya, Nepal, Senegal, Uganda and the United Republic of Tanzania that we analysed came from samples that were considered to be nationally representative of the countries’ health systems. The data from Haiti, Malawi, and Namibia came from service provision assessments that covered all of the health facilities in the countries; the data from Rwanda covered nearly all health facilities, with a partial sample of private facilities with fewer than five staff. The data from Bangladesh came from a sample that was considered nationally representative of all public facilities and all private hospitals, but excluded small private facilities. Although each assessment was based on a questionnaire for the facility manager, the manager’s responses were verified, whenever possible, by direct observation of the available infrastructure and supplies.

#### Readiness index calculation

The core of each DHS service provision assessment was a facility audit – i.e. a standardized assessment of the readiness of each surveyed facility to provide essential health services. The audit that was employed varied slightly between countries and was substantially revised, in 2012, to reflect a broader health system focus and to include health services for noncommunicable diseases.^24^ We extracted variables from the DHS service provision assessments to match as many as possible of the 50 items used in the formal assessment of the WHO service readiness index. Depending on the country involved, we calculated an index using between 37 and 49 of the 50 items (Table 1). In calculating each value of the index, we followed the WHO definition – i.e. we determined a percentage score for the items assessed in each of five domains and then calculated the mean of the resultant five scores to give an overall service readiness index for each facility.

#### Explanatory variables assessed

We assessed levels of association between the index and modifiable inputs to health-system performance^20^ in the domains of financing, organization and payment. The available data were, however, insufficient for us to identify potential determinants in the areas of behaviour and regulation. 

The available comparable national data on health-system financing included indicators of total health expenditure per capita, resources for health provided from sources external to the country – as a percentage of total health expenditure – and out-of-pocket expenditures on health – again as a percentage of total health expenditure. 

The available comparable facility data on the organization of the health system were whether the facility was a hospital or a health centre, clinic or dispensary and whether it was publicly or privately managed. 

Data on payment included the methods used to transfer money to health-care providers, such as user fees and donor funding to a facility. 

In addition, we investigated the relationship between the service readiness index and the land area of each study country – as a non-modifiable factor that could partially explain health-system readiness. Continuous covariates were natural-log-transformed. 

### Statistical analysis

Our analysis proceeded in three steps: (i) description of service readiness; (ii) comparison of readiness by facility type; and (iii) assessment of the potential determinants of service readiness.

We conducted descriptive analyses separately for hospitals and all other facilities. Within each study country, we calculated a mean service readiness index for the surveyed hospitals and a corresponding mean value for the surveyed health centres/clinics. We calculated intraclass correlation coefficients to compare the between-country variance in the values of the index with the corresponding within-country variance.

To compare readiness by facility type, we plotted service readiness for the surveyed private and public facilities – and the surveyed rural and urban facilities – within each country. To test for differences in the service readiness index between facility type, we used linear regression, with country as the stratifying variable.

To assess potential determinants of service readiness we limited the sample to the nine study countries that were low- or lower-middle-income and excluded Namibia, which is an upper-middle-income country. We used linear regression. We regressed the service readiness index on each of the financing, organizational and payment factors we investigated – as well as on the logarithm of land area. We regressed the service readiness index on each of the four facility-level characteristics (hospital, private, donor funding and user fees) and the four national factors (log THE, log OOP, log external support for health and log land area) in separate models. All models were adjusted for survey year to account for temporal trends and the development of the methods used for the DHS service provision assessments. From these eight linear regression models, we selected all the factors that gave a *P*-value below 0.20 and used them to create a combined model.^25^ Each regression model took the general form as follows:

*Y =B_0_ + B_1_X + B_2 _(α) *+ *ε* (1)

where *Y* represents service readiness, *B_0_ is* the intercept (average service readiness for the reference group of facilities), *α *represents the survey year,  *X* is the facility-level or national factor of interest and *ε* is an error term.

By including interaction terms between hospital and each other factor in a single model, we assessed evidence in the data for differences between the service readiness index’s associations in hospitals and the corresponding associations in the health centres/clinics. We retained the interaction terms that gave *P*-values below 0.05 for the final model. To provide realistic and easily comparable estimates, coefficients for each continuous factor were transformed to express the estimated difference in the service readiness index for a 10% change in that factor. All regression models were limited to facilities with complete data. Each multicountry analysis was weighted using the sampling weights used in the DHS service provision assessments, but rescaled so that each country contributed equally to the analysis. We calculated confidence intervals (CI) based on standard errors that accounted for clustering by country. Unless indicated otherwise, a *P*-value of 0.05 or lower was considered indicative of a statistically significant difference.

## Results

Our 10 study countries represent a range of low- and middle-income economies of varying geographical size and development status (Table 2). At the time of the DHS service provision assessments providing our data, mean life expectancies at birth ranged from 54 years in Uganda – in 2007 – to 71 years in Bangladesh – in 2014. In all of our study countries except Bangladesh and Nepal, these mean life expectancies were lower than the global mean values for low- and middle-income countries – which increased from 68 to 70 years between 2007 and 2015.^26^ At the time of the DHS service provision assessments, gross domestic products per capita varied from a low of 341 United States dollars (US$) in Malawi to a high of US$ 4134 in Namibia. At the same time, health-system funding varied. Median total annual health expenditure per capita was US$ 43. Some countries were characterized by high out-of-pocket expenditures on health – e.g. Bangladesh, where such expenditure represented 67% of total health expenditure – whereas others showed a heavy reliance on external funds – e.g. Malawi, where such funds represented 69% of total health expenditure.

**Table 2:** Characteristics of 10 countries in study sample, 2007–2015

Country	Year of SPA	No. of facilities surveyed			GDP per capita (US$)**^a^**	THE per capita (US$)**^a^**	OOPE (% of THE)**^a^**	ERFH (% of THE)**^a^**	Life expectancy at birth (years)**^a^**	Area (km^2^)**^b^**	Mean SRI (%)**^c^**	
		All	HC/C	Hospitals							HC/C	Hospitals
Bangladesh^d^	2014	1548^d^	1381	167	1087	31	67	12	71.2	130 000	41	74
Haiti^e^	2013	905^e^	784	121	810	65	32	31	62.4	28 000	52	73
Kenya^f^	2010	695	443	252	992	39	50	35	58.7	569 000	55	78
Malawi^e^	2013	977^e^	861	116	341	26	11	69	61.5	94 000	55	80
Namibia^e^	2009	411^e^	366	45	4124	332	9	12	61.4	823 000	68	82
Nepal^f^	2015	963	716	247	744	40	48	13	70.0	143 000	44	76
Rwanda^g^	2007	538^e^	496	42	398	34	25	52	57.9	25 000	59	79
Senegal^f,h^	2012−2014	727^d^	657	70	1051	47	39	27	66.4	193 000	60	69
Uganda^f^	2007	491	372	119	410	48	42	22	53.7	201 000	41	76
United Republic of Tanzania^f^	2015	1188	932	256	865	52	23	36	65.5	886 000	48	82
Full sample		8443	7008	1435							52	77

ERFH: external resources for health; GDP: gross domestic product; HC/C: health centres/clinics; OOPE: out-of-pocket expenditure; SPA: service provision assessment; THE: total health expenditure; US$: United States dollars.

^a^ For closest year available to year of service provision assessment.

^b^ To the closest 1000 km^2^.

^c^ The corresponding intraclass correlation coefficients were 0.29 for health centres/clinics and 0.09 for hospitals. The mean service-readiness-index values recorded for all facilities, health centres/clinics and hospitals were 54% (standard deviation, SD:17), 77% (SD:12) and 52% (SD:16), respectively.

^d^ Data came from sample considered representative of the country’s health system, excluding small private facilities.

^e^ Sample is a census of health facilities

^f^ Data came from sample considered representative of the country’s health system.

^g^ Data came from a census of public facilities and of private facilities with at least 5 staff plus a sample of small private facilities

^h^ Sample collected over 2 waves representing the first 2 years of a continuous 5-year assessment

Data sources: World Bank Global Development indicators,^26^ World Health Organization Global Health Observatory^27^ and authors’ analysis of data from service provision assessments.

In the DHS service provision assessments that provided our data, 8606 (97%) of the 8881 facilities sampled were considered to have been successfully surveyed. Of these, 163 (2%) were outreach facilities assessed with a shortened questionnaire; our analysis was confined to data from the 8443 facilities that each completed the full survey questionnaire (Table 2). At national level, the mean service readiness index for health centres/clinics ranged from 41% in Bangladesh and Uganda to 68% in Namibia, whereas the corresponding values for hospitals ranged from 69% in Senegal to 82% in both Namibia and the United Republic of Tanzania (Table 2). The index varied much more within countries than between countries. The within-country differences were the cause of 71% and 91% of the variation seen in the values for health centres/clinics and hospitals, respectively. Overall, although they still fell short of complete readiness for basic services, hospitals demonstrated higher service readiness than health centres/clinics (mean service readiness index: 77% versus 52%). 

Of the 8443 facilities included in our analysis, 636 (8%) were hospitals. Compared with the health centres/clinics, hospitals were more likely to be privately managed (53% versus 30%), to be in urban areas (73% versus 26%) and to receive financial support from donor organizations (37% versus 20%) and user fees (84% versus 58%) (Table 3). Over 75% (2052 of 2673) of the private facilities charged user fees – in comparison with about half (2957 of 5699) of the public facilities.

**Table 3:** Characteristics and service readiness of health facilities in study sample, 10 countries, 2007–2015

Characteristic	No. (%)**^a^**			No. of facilities with relevant data available
	All facilities (*n* = 8443)	Health centres/clinics (*n* = 7807)	Hospitals (*n* = 636)	
Hospital as facility type	636 (8)	0 (0)	636 (100)	8443
Private sector	2676 (32)	2335 (30)	340 (53)	8443
Urban^b^	1238 (29)	1017 (26)	221 (73)	5345
Facility income includes donor support^c^	1714 (21)	1483 (20)	232 (37)	8223
Facility income includes user fees for services	4972 (60)	4440 (58)	532 (84)	8372
Facility attained 100% service readiness index:				
For basic amenities	339 (4)	148 (2)	191 (30)	8443
For basic basic equipment	2269 (27)	1986 (25)	283 (44)	8443
For basic infection prevention	1012 (12)	836 (12)	176 (28)	8443
For basic diagnostic capacity	199 (2)	111 (1)	88 (14)	8443
For basic essential medications	151 (2)	51 (1)	100 (16)	8443
For overall service readiness	1 (0)	0 (0)	1 (0)	8443

^a^ The percentages shown are based on the sample-weighted numbers of facilities for which the relevant data were available and not, always, on the numbers surveyed.

^b^ The identification of a facility as urban or rural only occurred in the service provision assessments for five of the study countries: Bangladesh, Haiti, Malawi, Senegal and the United Republic of Tanzania

^c^ In the assessments in nine of the study countries, donor support was defined as support from secular or faith-based organizations or other unspecified donors. In the assessment in Nepal, however, it was defined as support from sources other than government ministries, user fees and training colleges.

The number of facilities with complete readiness in any one of the five domains investigated was low (Table 3). About a third (191/636) of the hospitals, but only 2% (148/7807) of the health centres/clinics appeared to have full service readiness in terms of basic amenities. Only 199 (2%) and 151 (2%) of the facilities we investigated had all of the diagnostics and essential medications that could be assessed in our calculations of the service readiness index, respectively. Although service readiness appeared generally better in the hospitals than in the smaller facilities, most of the hospitals still appeared inadequate in terms of basic amenities, diagnostic capacity and essential medications. Of the 8443 facilities sampled, just one had a perfect overall service readiness index of 100%. The assessed items that were most likely to be unavailable, even in the higher-performing facilities, were medications and diagnostics for noncommunicable diseases – e.g. haemoglobin tests, inhalers and statins.

**Fig. 1:**
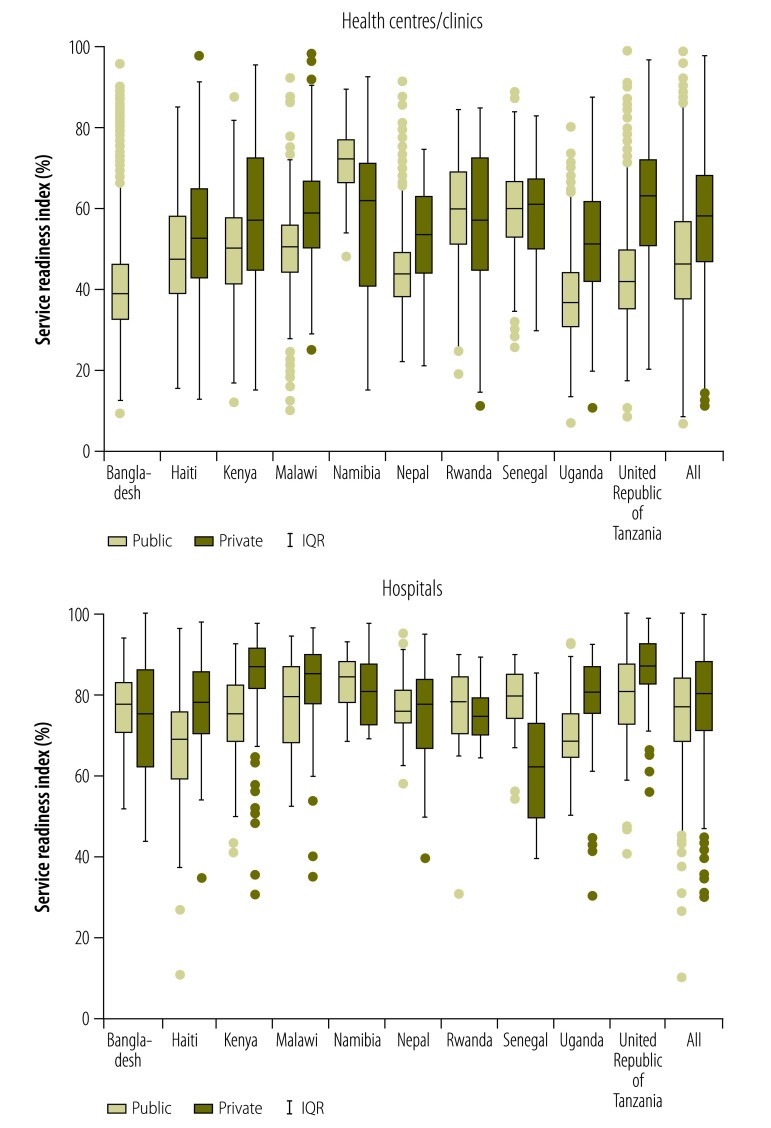
Differences between the service readiness indexes of private and public health facilities, ten countries, 2007–2015

**Fig. 2:**
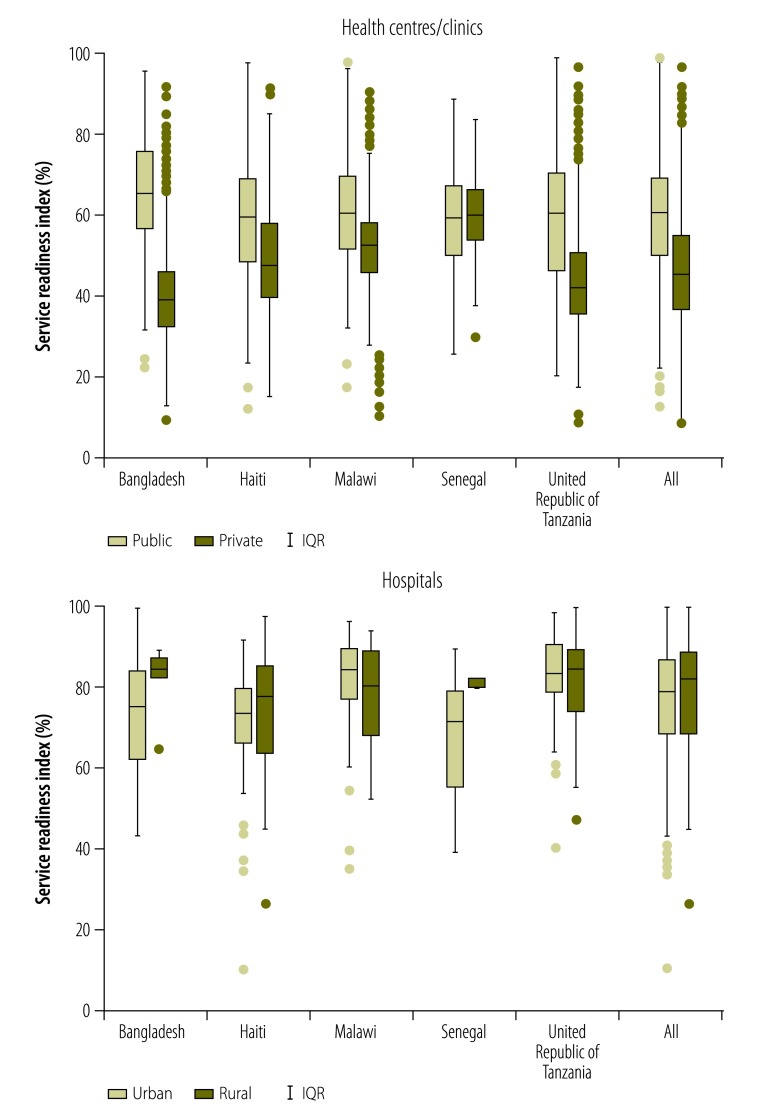
Differences between the service readiness indexes of rural and urban health facilities, five countries, 2012–2015

Fig. 1 depicts the medians and interquartile ranges for the values of the service readiness index recorded in public and/or private facilities in each of the 10 study countries and in the full sample. Compared with the median values for the public health centres/clinics, those for private health centres/clinics were significantly lower in Namibia, similar in Rwanda and Senegal and significantly higher in Haiti, Kenya, Malawi, Nepal, Uganda and the United Republic of Tanzania; Bangladesh was excluded from this comparison because small private clinics were not surveyed in that country’s DHS service provision assessment. Compared with the median values for the public hospitals, those for private hospitals were significantly higher in Haiti, Kenya, Malawi, Uganda and the United Republic of Tanzania but significantly lower in Senegal. Fig. 2 illustrates – for the five countries for which the relevant information was available – the differences in the service readiness index between urban and rural facilities. Urban health centres scored significantly higher than rural health centres in Bangladesh, Haiti, Malawi and the United Republic of Tanzania but not in Senegal. Urban hospitals had a similar service readiness index to rural hospitals in Haiti, Malawi and the United Republic of Tanzania, but had a significantly lower index than rural hospitals in Bangladesh and Senegal.

IQR: interquartile range.

Notes: Private health centres and clinics were not sampled in the Bangladesh service provision assessment. The boxplots reflect the range of the service readiness index in the observed data. In each boxplot, the horizontal line represents the median service readiness in public or private facilities, the box indicates the first and third quartiles, the whiskers extend to the observed value closest to 1.5 times the interquartile range below the 25^th^ percentile or above the 75^th^ percentile, and the points represent observed values outside the interval.

IQR: interquartile range.

Notes: These box-and-whisker plots summarize the data for just five study countries. Only the service provision assessments for these countries included records of the urban or rural location of each surveyed facility. The boxplots reflect the range of the service readiness index in the observed data. In each boxplot, the horizontal line represents the median service readiness in rural or urban facilities, the box indicates the first and third quartiles, the whiskers extend the observed value closest to 1.5 times the interquartile range below the 25^th^ percentile or above the 75^th^ percentile, and the points represent observed values outside the interval.

In the analysis of financial, payment and organizational factors associated with the service readiness index across the nine study countries that were low- or lower-middle-income, the charging of user fees, hospital as facility type, the percentage of total health expenditure represented by external contributions and private management each explained at least 10% of the variance seen in separate models adjusted only for survey year (Table 4). The fully adjusted model explained 34% of the total variance seen in the service readiness index. In the same model, hospitals had a significantly higher service readiness index than health centres/clinics (linear increase: 21.5%; 95% CI: 16.9 to 26.2). If other factors were held constant, each 10% increase in the percentage of total health expenditure represented by external contributions was associated with a difference of 0.7 of a percentage point in the service readiness index (95% CI: −0.3 to 1.7). In hospitals the association of facility funding sources – i.e. user fees and donor support – with the index differed significantly from that calculated for health centres/clinics. In the model with interaction terms for these factors, the estimated service readiness index was 53% for health centres/clinics with user fees and 46% for health centres/clinics without such fees. The corresponding values for hospitals were 73% and 72%, respectively. Donor funding was associated with estimated linear increases in the service readiness index of 3.0 percentage points in health centres/clinics and 4.4 percentage points in hospitals.

**Table 4:** Association of health system and facility characteristics with the facility service readiness index, nine countries, 2007–2015

Characteristic	Separate models adjusted for survey year only**^a^**				Adjusted model**^a^**	
	Estimated difference in SRI (95% CI)	*P*	R^2^		Estimated difference in SRI (95% CI)	*P*
**Facility**						
Hospital as facility type	25.8 (19.4 to 32.2)	< 0.01	0.17		21.5 (16.9 to 26.2)	< 0.01
Charges user fees	12.6 (5.6 to 19.7)	< 0.01	0.15		6.7 (−0.3 to 13.7)	0.06
Receives donor funding	9.5 (4.9 to 14.2)	< 0.01	0.07		3.2 (1.3 to 5.1)	< 0.01
Privately managed	11.1 (2.9 to 19.3)	0.01	0.11		5.2 (−1.4 to 11.8)	0.11
**National or survey**						
THE per capita^b^	−0.1 (−1.8 to 1.7)	0.94	0.01		NI	N/A
% of THE represented by external support for health system^b^	0.9 (0.3 to 1.5)	0.01	0.11		0.7 (−0.3 to 1.7)	0.13
% of THE represented by out-of-pocket expenses^b^	−0.7 (−1.2 to −0.1)	0.04	0.05		0.3 (−0.6 to 1.2)	0.46
Land area of country^b^	−0.1 (−0.5 to 0.2)	0.29	0.02		NI	N/A
Survey year	−0.5 (−2.6 to 1.7)	0.64	0.01		0.1 (−1.2 to 1.4)	0.87

CI: confidence interval; N/A: not applicable; NI: not included; SRI: service readiness index; THE: total health expenditure.

^a^ The models were based on data from service provision assessments in 7851 health facilities. All of the models were weighted using sampling weights scaled so that each study country contributed equally. The adjusted model, which contained covariates found to give *P*-values below 0.2 in the separate models, gave an R^2^ value of 0.34.

^b^ The estimated difference represented a 10% increase in this characteristic.

## Discussion

The aim of our study was to improve our understanding of the extent to which service readiness of health facilities varies within and across low- and middle-income countries. This work updates and expands upon the prior comparative assessment of service readiness^14^and highlights opportunities for improving health infrastructure at the start of the era of the SDGs. Our cross-country comparison of structural readiness in over 8000 health facilities revealed substantial and pervasive gaps in the basic capacity to provide health-care services. In general, health centres/clinics achieved barely over half – and hospitals just over three quarters – of the maximum possible score for service readiness. Although the service readiness index defines the resources that WHO hopes to see in any facility providing health-care services, only one assessed facility had all of those resources. In all of the study countries, gaps were particularly notable in single-use items such as medications and diagnostics. Small, multi-use items, such as basic equipment, were more prevalent. These patterns are similar to those seen in a previous, but smaller cross-country comparison of service readiness.^14^

While mean readiness differed among the countries in the study, the variation between facilities in the same country was much larger. Although all of the study countries appeared to be able to equip some facilities well, all of them were also failing to ensure consistent readiness throughout their health systems. Public health centres/clinics and rural health centres/clinics were particularly low-performing in many countries – but not all. Previous research in individual countries has suggested that, compared with the private sector, the public sector provides care of higher quality in some countries and care of lower quality elsewhere.^28^^–^^30^ Our analyses, using a standard measure and data that are nationally representative, not only led to a similar observation but also indicate that, within any given country, the trend for the public sector to appear better –or worse – than the private can depend on facility type. In identifying the delivery of adequate care in rural areas as being a major challenge – because of major deficiencies in equipment and supplies – our findings support the results of earlier, single-country studies.^31^^,^^32^

Compared with health centres/clinics, we found that hospitals appeared to have much better service readiness, especially in terms of basic amenities such as water and electricity supplies. This finding and the similar deficits identified in a study of maternal care in facilities without surgical capacity^33^ raise concerns about the general readiness of primary care facilities. Primary care is an important source of essential health care – including for underserved populations. Such care could be a powerful platform for responding to a range of health challenges in lower-income countries^34^^,^^35^ – but only if we give increased attention to the infrastructure and supplies at the health centres and clinics that serve as the first line of care for populations, particularly in rural areas.

Our analysis of some potential determinants of service readiness yielded unexpected results. While external support for the health system was positively associated with the mean values of the service readiness index, none of the elements of national financing tested in our analysis demonstrated a strong relationship with health-facility readiness. Variation within the nine countries included in this particular analysis may have overwhelmed any underlying causal relationships. Such variation in health-system capacity may explain why countries with similar incomes and epidemiological burdens have achieved very different health outcomes.^21^^,^^36^^–^^40^ The associations we observed between facility payment factors and service readiness reinforce the idea that, in any given country, health-system performance may be more greatly improved by reducing the variability between facilities than by simply increasing health funding at the national level. Research on within-country changes in health-system inputs and on the resulting capacity is needed. The results of our analyses indicate specific areas for follow-up research, such as the relatively strong performance of public health facilities – compared with private ones – in Namibia and Senegal.

This research has several limitations. For example, depending on the country, no information on one to 13 items, of the 50 used in the formal assessment of the service readiness index, was available. The older DHS service provision assessments we considered did not assess medications for noncommunicable diseases. Lack of information on some items made it more difficult to gauge the full service readiness in some countries. However, our calculations of the service readiness index excluded these items and also controlled for survey year – to account for the temporal differences in the types of data recorded in the DHS service provision assessments. Although the assessment in Bangladesh excluded small private facilities, all of the other assessments were based on nationally representative samples or were complete – or nearly complete – censuses of all health facilities. To maximize sample size and comparability, we selected all the countries in which DHS service provision assessments had been conducted in the decade preceding our analyses. The inclusion of additional countries could provide greater capacity to test the contribution of national factors to facility readiness.

The main aims of the present study were to describe service readiness, to compare it across key strata within countries and to assess potential determinants across countries. While our findings are not generalizable to all low- and middle-income countries – particularly those in regions not covered in the sample, such as Latin America and East Asia – they cover seven of 31 low-income countries in the world. Taken together with prior research on service readiness^14^ and process quality^5^^,^^41^ in other lower-income countries, the results highlight the depth and breadth of the quality deficits in health care that must be addressed on the road to universal health coverage. Efforts to ensure access to health care will fail to improve population outcomes if health facilities lack the basic capacity to provide care. Although the service readiness index may be used to assess the quality of infrastructure and equipment, it cannot indicate the provision of competent care. The provision of such care is likely to be even rarer than good service readiness.^5^^,^^42^^,^^43^ The health systems in all 10 of our study countries experience shortages in basic resources for essential services. For these countries, efforts at quality improvement must be national in scope and, in most cases, designed with a specific focus on addressing the inequities for those accessing rural and/or public health centres/clinics. Countries may benefit from identifying the best-performing facilities as case studies for better practices and from working to standardize support for service readiness nationwide. More broadly, reducing variability and improving efficiency in the translation of health-system inputs to facility readiness – and, ultimately, the translation of readiness to the quality of care delivered – are critical steps in the pathway to health care that is both available and effective.
